# Supplementary material for: α-Synuclein fibrils enhance HIV-1 infection of human T cells, macrophages and microglia
Source: Nat Commun. 2025 Jan 18;16:813. doi: 10.1038/s41467-025-56099-z (PMC11742913; doi:10.1038/s41467-025-56099-z)
Supplement: Supplementary file 1 — Supplementary Information [file 41467_2025_56099_MOESM1_ESM.pdf]

Supplementary Materials for:

α-Synuclein fibrils enhance HIV-1 infection of human T cells, macrophages and microglia

Olari, Liu *et al.*

*Corresponding author. Email: frank.kirchhoff@uni-ulm.de

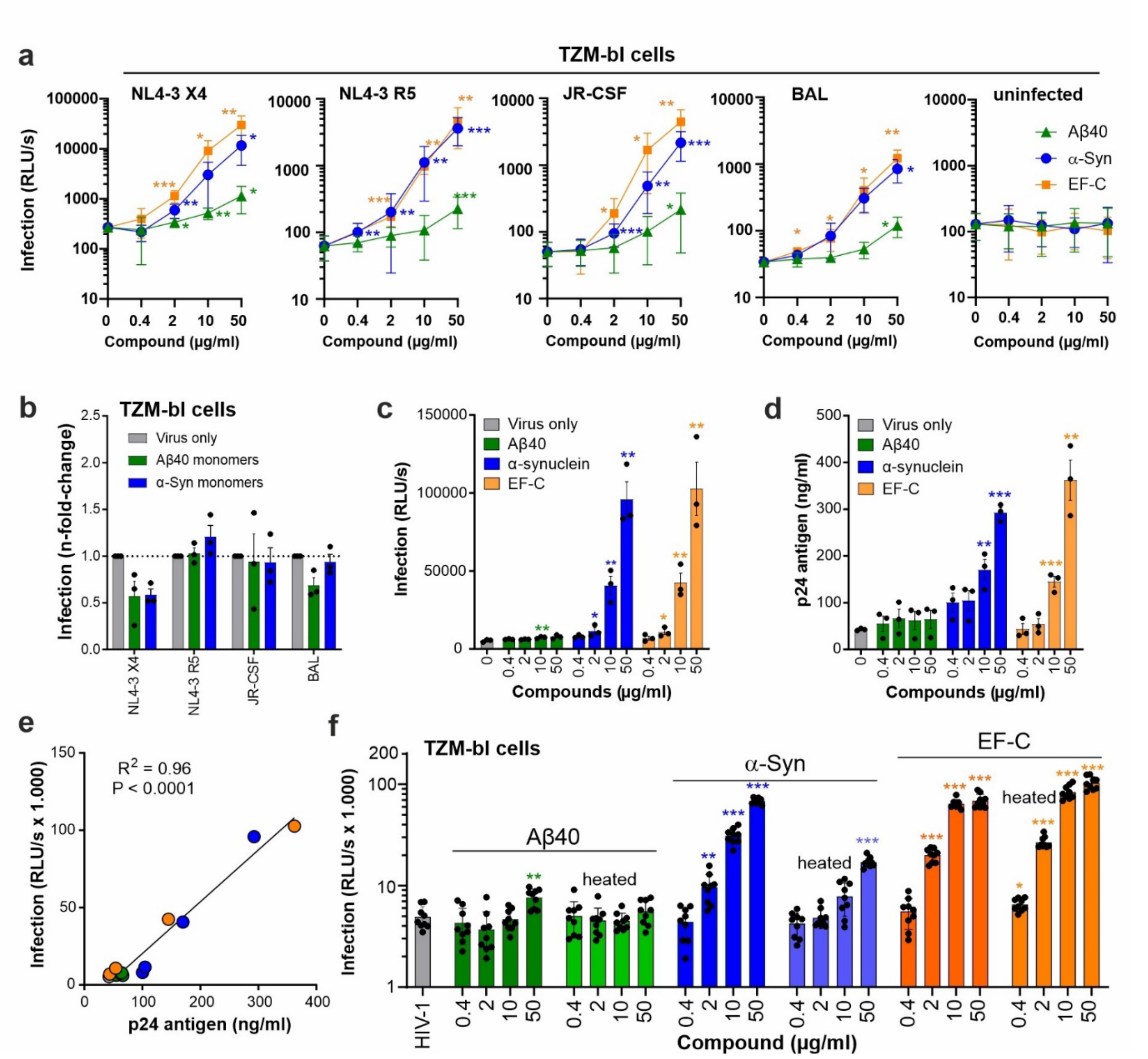

Supplementary Fig. 1. Effects of Aβ40, α-synuclein, and EF-C fibrils on HIV-1 Infection. **(a)** TZM-bl cells were infected with the indicated HIV-1 strains, briefly exposed to different doses of Aβ40, α-synuclein (α-Syn), or EF-C fibrils, or left untreated. Infection was quantified three days post-infection by detecting the expression of β-galactosidase (relative light units per second, RLU/s) in the TZM-bl cells. In the right panel, values obtained for cells that were left uninfected but treated with the different amyloids are shown. **(b)** TZM-bl cells were infected as in panel a, except that monomeric peptides were used for virion treatment. (**c-e**) R5-tropic HIV-1 NL4-3 pre-incubated with the indicated concentrations of fibrils was used to infect TZM-bl cells. Three days later, infections rates were determined by β-galactosidase reporter assay **(c)** and virus production by p24 ELISA **(d)**. Panel **e** shows the correlation between the results obtained using the two assays. **(f)** TZM-bl cells were infected as described in panel a, except that untreated and heat-treated Aβ40, α-synuclein (α-Syn), and EF-C fibrils were used for the treatment of R5-tropic NL4-3 virions. Significant differences in all panels were determined using two-sided unpaired t-test analysis. Asterisks indicate statistical significance (**P*<0.05, ***P*<0.01, ****P*<0.001). Source data are provided as a Source Data file.

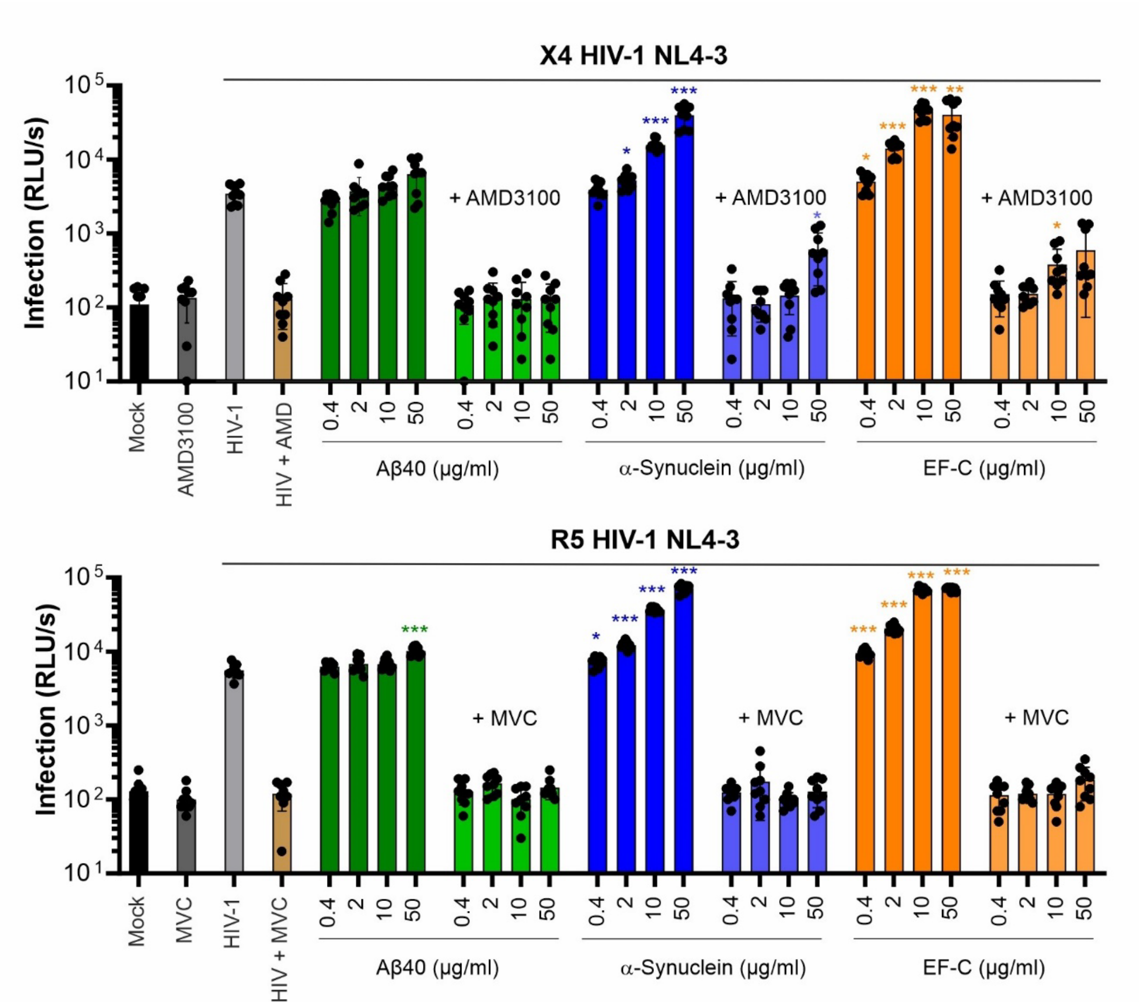

Supplementary Fig. 2. Effect of antiretroviral drugs on HIV-1 Infection in the presence of Aβ40 and α-synuclein fibrils. TZM-bl cells were pre-treated with AMD3100 (200 nM) or Maraviroc (MVC, 500 nM) for 1 h at 37 °C. Aβ40, α-synuclein, and EF-C fibrils were then pre-incubated with HIV-1 NL4-3 X4 (upper panel) or HIV-1 NL4-3 R5 (lower panel) and added to the cells. Infection was quantified three days post-infection by detecting the expression of β-galactosidase (relative light units per second, RLU/s) in the TZM-bl cells. Significant differences were determined using two-way ANOVA with Dunnett´s multiple comparison test. Asterisks indicate statistical significance (**P*<0.05, ***P*<0.01, ****P*<0.001). Source data are provided as a Source Data file.

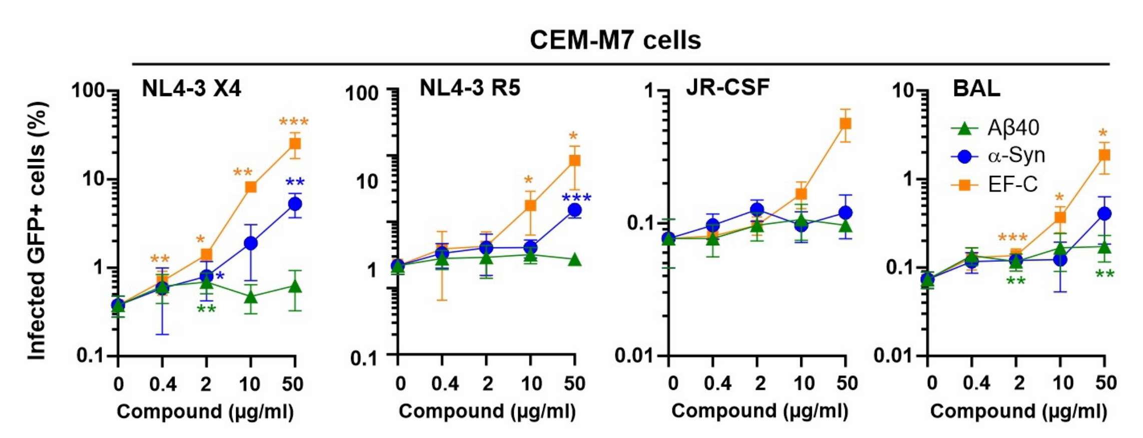

Supplementary Fig. 3. Effect of Aβ40 and α-synuclein fibrils on HIV-1 Infection in CEM-M7 cells. Aβ40, α-synuclein (α-Syn), and EF-C fibrils were pre-incubated with indicated HIV-1 strains and added to CEM-M7 cells. Infection was quantified three days post-infection by quantifying the GFP+ cells by flow cytometry. Significant differences were determined using two-sided unpaired t-test analysis. Asterisks indicate statistical significance (*$P<0.05$, **$P<0.01$, ***$P<0.001$). Source data are provided as a Source Data file.

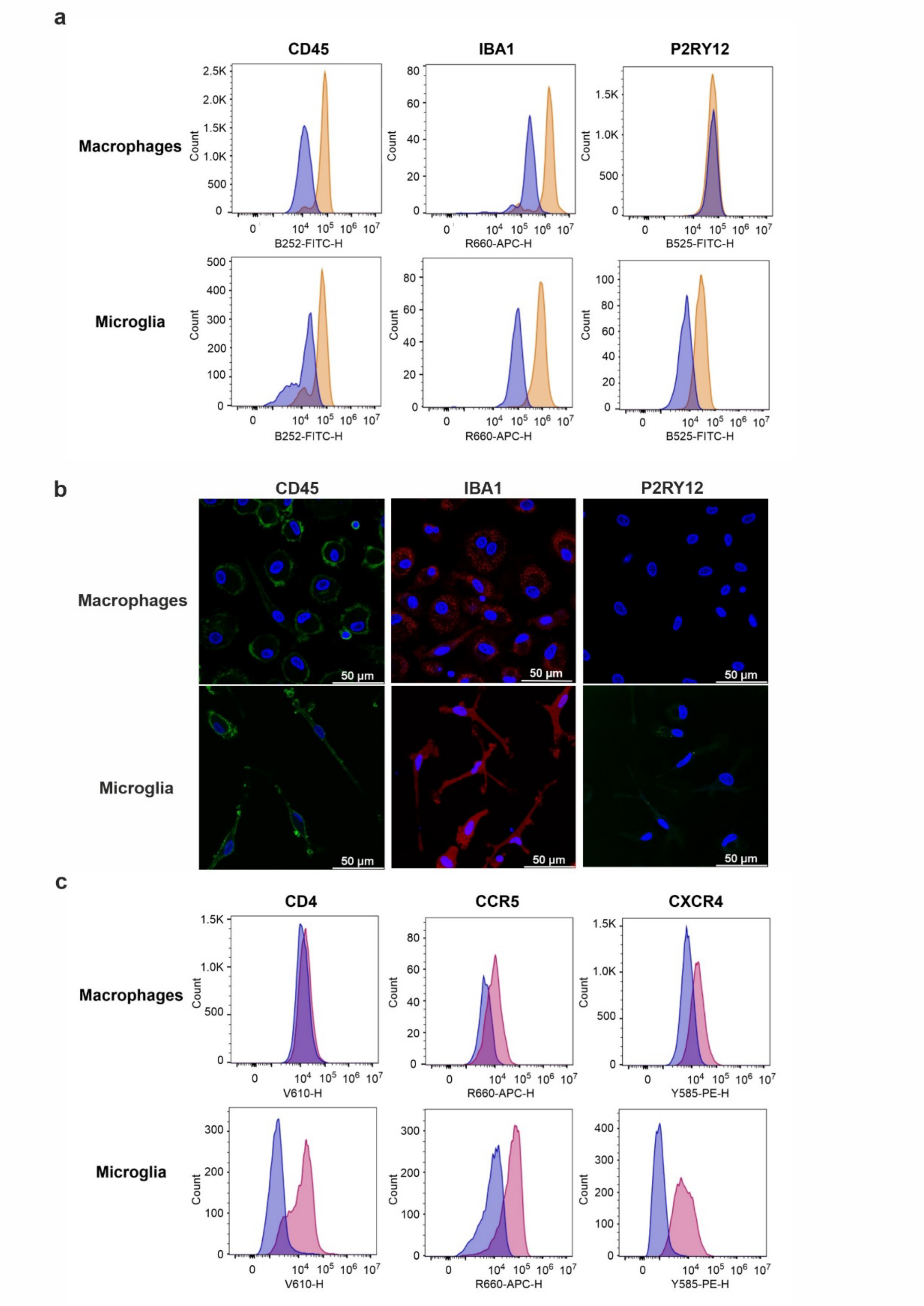

Supplementary Fig. 4. Characterization of PBMC-derived macrophages and microglia. **(a, b)** Evaluation of myeloid cell markers CD45, IBA1, and P2RY12 expression by **(a)** flow cytometry and **(b)** confocal microscopy. In **(a)**, the antibody isotype controls staining histograms (blue) are overlaid with the antibody staining (orange). Images were taken with a Leica DM8i confocal microscope (Leica). **(c)** Evaluation of HIV-1 receptor CD4 and co-receptors CCR5 and CXCR4 expression by flow cytometry. Antibody isotype control staining histograms (blue) are overlaid with the antibody staining (pink).

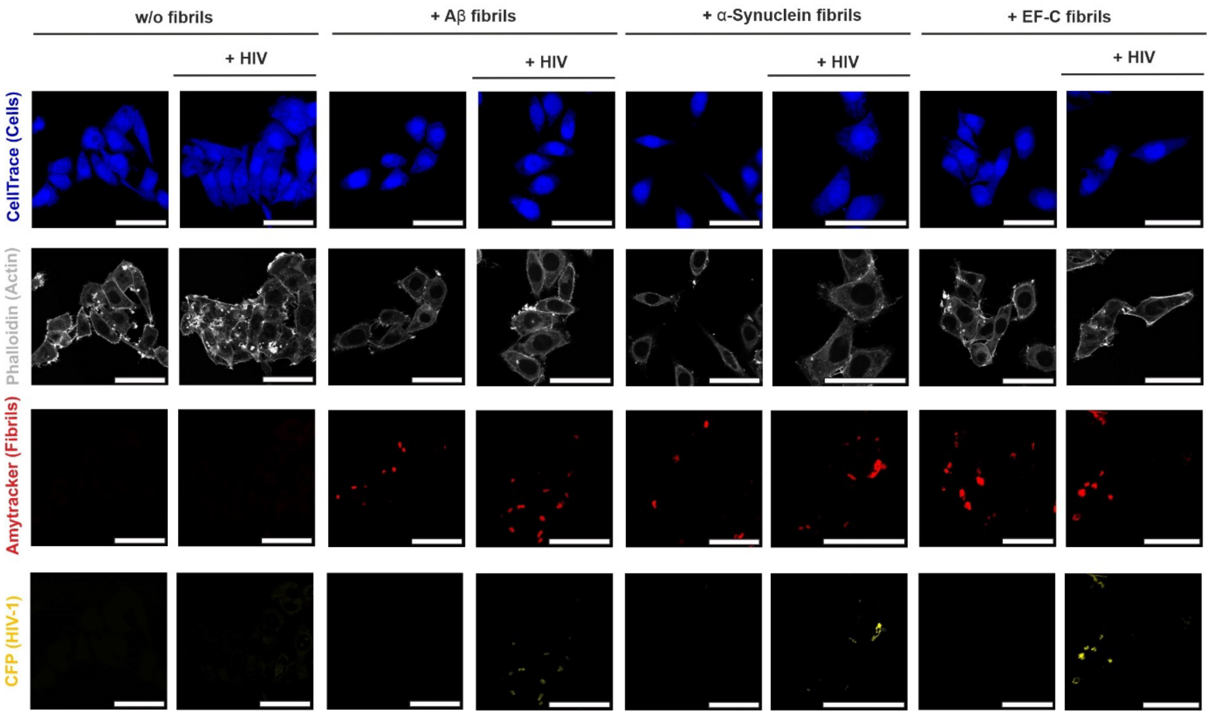

Supplementary Fig. 5. Imaging of HIV-1 particles and their target cells in the presence and absence of amyloid fibrils. Fluorescence microscopy images showing Aβ40, α-synuclein (α-Syn), and EF-C fibrils that were stained with Amytracker 540 dye (red) and CFP labeled HIV (yellow) in the absence and presence of TZM-bl cells stained with the cytoskeleton dye (CellTrace) (blue) and actin dye (ATTO-phalloidin) (white). Scale bars are 50 µm. Merges and enlargements are shown in Figure 5**a**. Images were taken with an LSM 710 confocal microscope (Zeiss).

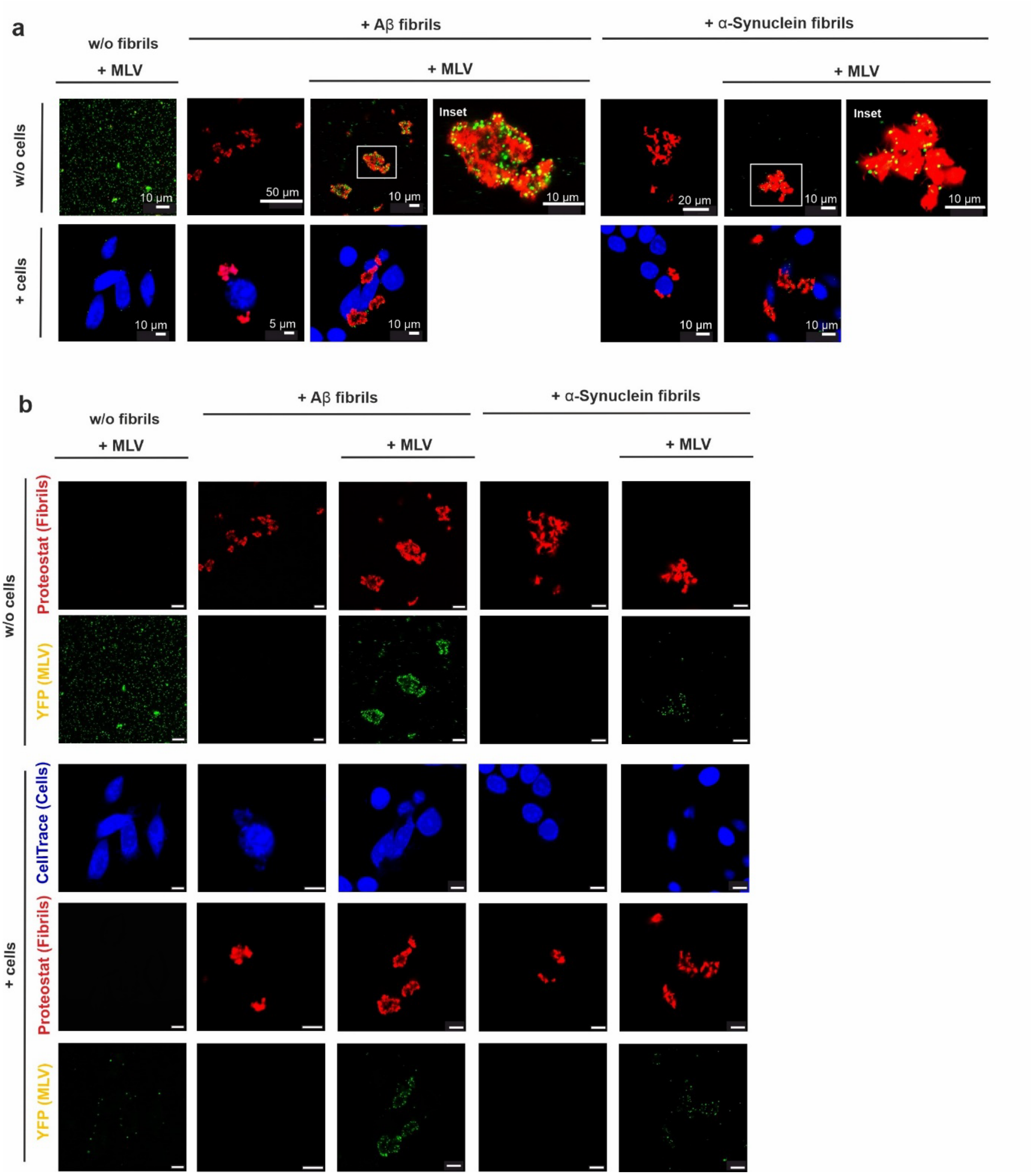

Supplementary Fig. 6. Aβ42 and α-synuclein fibrils promote attachment of MLV particles to target cells. (a) Fluorescence microscopy images showing Aβ42 and α-synuclein fibrils that were stained with ProteoStat amyloid dye (red) and YFP labeled murine leukemia virus (MLV) (green) in the absence and presence of TZM-bl cells stained with cytoskeleton dye (CellTrace) (blue). Images were taken with an LSM 710 confocal microscope (Zeiss), and scale bars are indicated in each image. (**b**) Fluorescence microscopy imaging as in (**a**) but showing all channels and conditions individually. Scale bars are 10 µm. Images were taken with an LSM 710 confocal microscope (Zeiss).

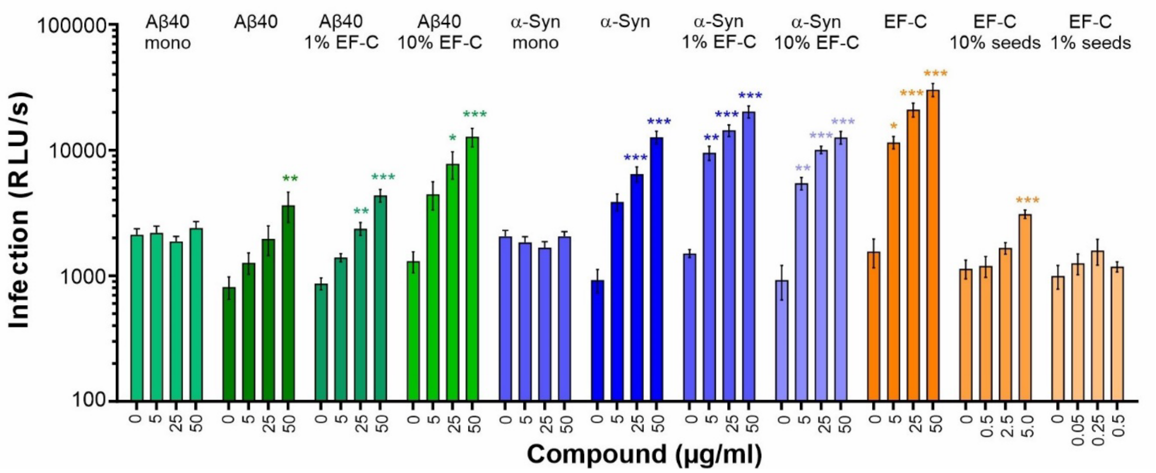

Supplementary Fig. 7. Effect of Aβ40 and α-synuclein fibrils cross-seeded by HIV-1 Env-derived EF-C peptide on HIV-1 infection. Monomeric (mono) or pre-formed Aβ40 and α-synuclein fibrils cross-seeded with 10% or 1% EF-C seeds were pre-incubated with HIV-1 NL4-3 R5-tropic virus and added to TZM-bl cells. Infection was quantified three days post-infection by detecting the expression of β-galactosidase (relative light units per second, RLU/s). Values were corrected for the background signal derived from the uninfected cells. Shown is the mean of three independent experiments measured in triplicates ± SEM. Significance was determined using two-sided unpaired t-test analysis. Asterisks indicate statistical significance (*P<0.05, **P<0.01, ***P<0.001). Source data are provided as a Source Data file.

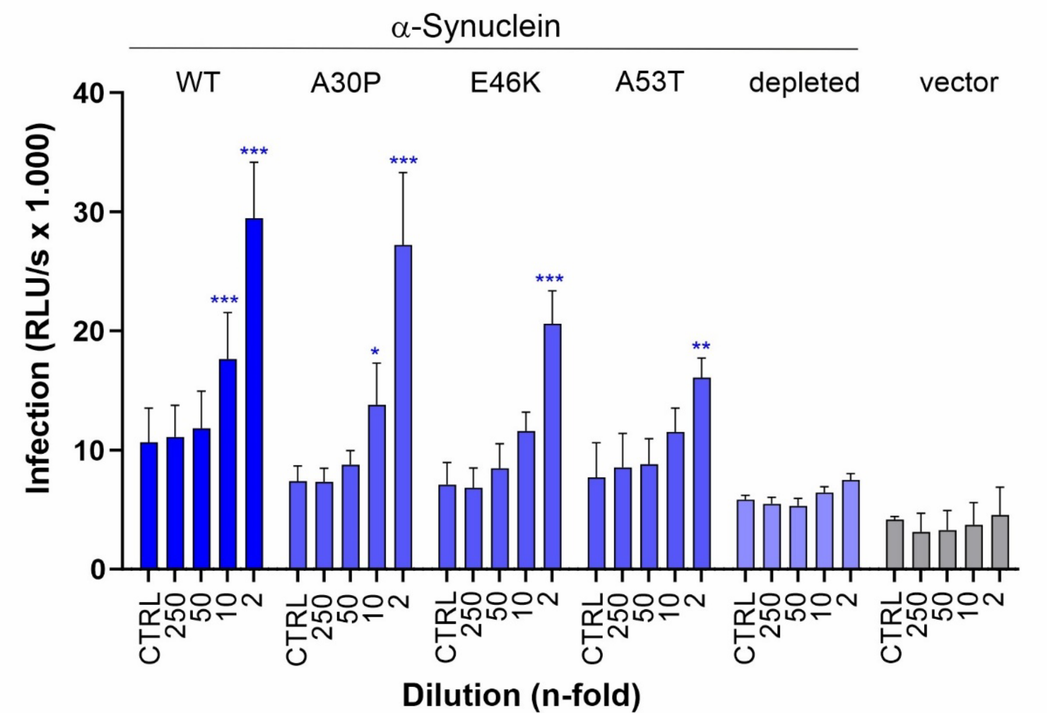

Supplementary Fig. 8. α-synuclein released from cells enhances HIV-1 infection. Cell supernatants containing α-synuclein oligomers were generated upon transfection of H4 cells with indicated α-synuclein constructs containing a split Gaussia luciferase construct or with an empty vector. α-synuclein was depleted in the supernatant of wild-type transfected H4 cells using magnetic protein G beads and SYN-1 antibody as described in the method section. Supernatants were diluted in PBS, pre-incubated with HIV-1 AD8, and added to TZM-bl cells. Infection was quantified three days post-infection by detecting the expression of β-galactosidase (relative light units per second, RLU/s) in the TZM-bl cells. Values were corrected for the background signal derived from the uninfected cells. Shown is the mean of three independent experiments measured in triplicates ± SEM. Significant differences were determined using two-way ANOVA with Dunnett´s multiple comparison test. Asterisks indicate statistical significance (**P*<0.05, ***P*<0.01, ****P*<0.001). Source data are provided as a Source Data file.

Supplementary Table 1. Human brain lysates analyzed.

Sample	Age	Gender	Residency	Diagnosis	Braak -LBD	Braak-NFT	Aβ Phase	CERAD	NIA-AA Degree of AD	PMI
AD	91	F	Germany	AD	n.d.	4	3	1	2	n. a
LBD	71	F	Germany	PART	4	1	0	0	0	72 h
AD, LBD	84	M	Germany	AD	6	6	4	3	3	120 h
HC1	62	M	Germany	PART	0	1	0	0	0	12 h
HC2	69	F	Germany	PART	0	1	0	0	0	24 h
HC3	66	M	Germany	PART	0	1	0	0	0	72 h

Post-mortem tissue was provided by the Laboratory of Neuropathology at Ulm University and neuropathologically characterized by Dr. Thal. Diagnosis: Alzheimer's disease (AD), healthy controls (HC); primary age-related tauopathy (PART); Braak Lewy body disease (LBD) stages (0-6)[1], Braak neurofibrillary tangles (NFT) stages (1-6)[2], Aβ phase (0-5)[3], and the CERAD (Consortium to Establish a Registry for Alzheimer's Disease) score for neuritic plaque density (0-3)[4]; NIA-AA degree of AD pathology (0-3)[5]; PMI (post-mortem interval of the tissue collection).

References

1. Braak, H. *et al.* Staging of brain pathology related to sporadic Parkinson's disease. *Neurobiol. Aging* **24**, 197–211 (2003).
2. Braak, H. & Braak, E. Neuropathological stageing of Alzheimer-related changes. *Acta Neuropathol. (Berl.)* **82**, 239–259 (1991).
3. Thal, D. R., Rüb, U., Orantes, M. & Braak, H. Phases of A beta-deposition in the human brain and its relevance for the development of AD. *Neurology* **58**, 1791–1800 (2002).
4. Mirra, S. S. *et al.* The Consortium to Establish a Registry for Alzheimer's Disease (CERAD). Part II. Standardization of the neuropathologic assessment of Alzheimer's disease. *Neurology* **41**, 479–486 (1991).
5. Montine, T. J. *et al.* National Institute on Aging-Alzheimer's Association guidelines for the neuropathologic assessment of Alzheimer's disease: a practical approach. *Acta Neuropathol. (Berl.)* **123**, 1–11 (2012).
