# Supplementary material for: α-Synuclein fibrils enhance HIV-1 infection of human T cells, macrophages and microglia
Source: Nat Commun. 2025 Jan 18;16:813. doi: 10.1038/s41467-025-56099-z (PMC11742913; doi:10.1038/s41467-025-56099-z)
Supplement: Supplementary file 2 — Reporting Summary [file 41467_2025_56099_MOESM2_ESM.pdf]

nature portfolio

Corresponding author(s): Frank Kirchhoff, ORCID: 0000-0002-7052-2360

Last updated by author(s): Dec 18, 2024

Reporting Summary

Nature Portfolio wishes to improve the reproducibility of the work that we publish. This form provides structure for consistency and transparency in reporting. For further information on Nature Portfolio policies, see our Editorial Policies and the Editorial Policy Checklist.

Statistics

For all statistical analyses, confirm that the following items are present in the figure legend, table legend, main text, or Methods section.

n/a	Confirmed	
☐	☒	The exact sample size (*n*) for each experimental group/condition, given as a discrete number and unit of measurement
☐	☒	A statement on whether measurements were taken from distinct samples or whether the same sample was measured repeatedly
☐	☒	The statistical test(s) used AND whether they are one- or two-sided<br>*Only common tests should be described solely by name; describe more complex techniques in the Methods section.*
☒	☐	A description of all covariates tested
☒	☐	A description of any assumptions or corrections, such as tests of normality and adjustment for multiple comparisons
☐	☒	A full description of the statistical parameters including central tendency (e.g. means) or other basic estimates (e.g. regression coefficient) AND variation (e.g. standard deviation) or associated estimates of uncertainty (e.g. confidence intervals)
☐	☒	For null hypothesis testing, the test statistic (e.g. *F*, *t*, *r*) with confidence intervals, effect sizes, degrees of freedom and *P* value noted<br>*Give P values as exact values whenever suitable.*
☒	☐	For Bayesian analysis, information on the choice of priors and Markov chain Monte Carlo settings
☒	☐	For hierarchical and complex designs, identification of the appropriate level for tests and full reporting of outcomes
☒	☐	Estimates of effect sizes (e.g. Cohen's *d*, Pearson's *r*), indicating how they were calculated

Our web collection on statistics for biologists contains articles on many of the points above.

Software and code

Policy information about availability of computer code

Data collection	Gen5 3.08; SoftMax 7.0.3, Black Zen Studio 2010, LasX 3.7.6, CytExpert 2.3, Simplicity 4.20, Zeta view 8.05
Data analysis	Corel DRAW 23.1, GraphPad Prism 10.3.1, FlowJo 10.9.0, CytExpert 2.3, Fiji 9.1, Microsoft Office Professional Plus 2019

For manuscripts utilizing custom algorithms or software that are central to the research but not yet described in published literature, software must be made available to editors and reviewers. We strongly encourage code deposition in a community repository (e.g. GitHub). See the Nature Portfolio guidelines for submitting code & software for further information.

Data

Policy information about availability of data

All manuscripts must include a data availability statement. This statement should provide the following information, where applicable:

- Accession codes, unique identifiers, or web links for publicly available datasets
- A description of any restrictions on data availability
- For clinical datasets or third party data, please ensure that the statement adheres to our policy

A data availability statement is included.

Research involving human participants, their data, or biological material

Policy information about studies with human participants or human data. See also policy information about sex, gender (identity/presentation), and sexual orientation and race, ethnicity and racism.

Reporting on sex and gender	From the 6 human brain tissues we analyzed, 3 were obtained from males, and 3 from females. No sex- and gender-based analysis were performed. The donors of the buffy coats were fully anonymized.
Reporting on race, ethnicity, or other socially relevant groupings	No race- or ethnicity-based analysis was performed.
Population characteristics	Human brain tissue was obtained from deceased individuals in the age range 62-91. From the 6 samples, 3 were from individuals with no diagnosed cognitive impairment, one patient was diagnosed with Alzheimer's disease, one patient with Lewy Body Disease, and one patient with both. More details regarding the diagnosis can be seen in the Supplementary Table 1.
Recruitment	n/a
Ethics oversight	The analysis of brain samples recieved ethical approval by the Ulm University Ethics Committee (Ulm/Germany; Decision-No. 342/14) and by the UZ Leuven ethical committee (Leuven/Belgium; Decision-No. S-59295). The use of human PBMCs was approved by the Ethics Committee of the Ulm University Medical Center (Approval 93/21-FSt/TR).

Note that full information on the approval of the study protocol must also be provided in the manuscript.

Field-specific reporting

Please select the one below that is the best fit for your research. If you are not sure, read the appropriate sections before making your selection.

☒ Life sciences ☐ Behavioural & social sciences ☐ Ecological, evolutionary & environmental sciences

For a reference copy of the document with all sections, see nature.com/documents/nr-reporting-summary-flat.pdf

Life sciences study design

All studies must disclose on these points even when the disclosure is negative.

Sample size	Sample sizes were not statistically assessed. Sample size was chosen based on previous experience and established protocols. Results were confirmed in three independent experiments, as indicated in the legends. One experiment was performed once in triplicates, due to the limited availability of the unique biological material.
Data exclusions	No data was excluded.
Replication	The number of independent replicates to similar results is indicated in the respective figure legends. All technically sound attempts at replication yielded similar results.
Randomization	Randomization was not applicable for this study, as no human trials or cohort studies were performed or samples assigned to experimental groups.
Blinding	Blinding was not applicable.

Reporting for specific materials, systems and methods

We require information from authors about some types of materials, experimental systems and methods used in many studies. Here, indicate whether each material, system or method listed is relevant to your study. If you are not sure if a list item applies to your research, read the appropriate section before selecting a response.

Materials & experimental systems

n/a	Involved in the study
☐	☒ Antibodies
☐	☒ Eukaryotic cell lines
☒	☐ Palaeontology and archaeology
☒	☐ Animals and other organisms
☒	☐ Clinical data
☒	☐ Dual use research of concern
☒	☐ Plants

Methods

n/a	Involved in the study
☐	☐ ChIP-seq
☐	☒ Flow cytometry
☐	☐ MRI-based neuroimaging

Antibodies

Antibodies used

Rabbit Recombinant Monoclonal Iba1 antibody - conjugated to Alexa Fluor® 647 Abcam Cat#ab225261 (1:100);
Alexa Fluor® 647 Rabbit IgG, monoclonal, Abcam Cat#199093 (1:100);
FITC anti-human CD45 Antibody, Biolegend Cat#304005 (1:100),
FITC Mouse IgG1, κ Isotype Ctrl Antibody, Biolegend Cat#400107 (1:100);
FITC anti-human P2RY12 Antibody; Biolegend Cat#392107, (1:100);
FITC Mouse IgG2a, κ Isotype Ctrl (FC) Antibody, Biolegend Cat#400209, (1:100);
Brilliant Violet 421™ anti-human CD4 Antibody, Biolegend Cat#317434, (1:100);
Brilliant Violet 421™ Mouse IgG2b, κ Isotype Ctrl Antibody, Biolegend, Cat#400342 (1:100);
BD Pharmingen™ APC Mouse Anti-Human CD195, BD Pharmingen, Cat#550856 (1:50);
BD Pharmingen™ APC Mouse IgG2a, κ Isotype Control, Cat#555576 (1:50);
BD Pharmingen™ PE Rat Anti-Human CD184, BD Pharmingen, Cat#551510 (1:200);
BD Pharmingen™ PE Rat IgG2a, κ Isotype Control, BD Pharmingen, Cat#554689 (1:200);
BD Transduction Laboratories™ Purified Mouse Anti-α-Synuclein, BD, Cat#610787 (1:20);
Anti-Flavivirus group antigen [D1-4G2-4-15 (4G2)], Absolute Antibody, Cat#Ab00230-2.0 (1:10000);
Goat anti-Mouse IgG (H+L) Secondary Antibody, HRP, Thermo Fischer Scientific, Cat#A16066, (1:20000);
Polyclonal rabbit antiserum against p24 (Eurogentec);
Goat IgG anti-Rabbit IgG (Fc)-HRPO, Dianova, Cat#111-035-008 (1:2000).

Validation

Abcam: Antibodies are validated in western blot using lysates from cells or tissues that we have identified to express the protein of interest. Once we have determined the right lysates to use, western blots are run and the band size is checked for the expected molecular weight. We will always run several controls in the same western blot experiment, including positive lysate and negative lysate. When possible, we also include knock-out (KO) cell lines as a true negative control for our western blots. We are always increasing the number of KO-validated antibodies we provide. In addition, we run old stock alongside our new stock. If we know the old stock works well, this also acts as a suitable positive control. If the western blot result gives a clear clean band and we are happy with the result from the control lanes, these antibodies will be passed and added to the catalog. Additionally, Abcam antibodies are checked using Biophysical Quality Control (QC) that enables confirmation of antibody identity at a molecular level.

Dianova: Validation based on immunoelectrophoresis and/or ELISA, the antibody reacts with the Fc portion of rabbit IgG heavy chain but not with the Fab portion of rabbit immunoglobulins.

Thermo Fischer Scientific: The sensitivity of each lot of antibody is confirmed using ELISA. The specificity of each lot of antibody is confirmed by immunoelectrophoresis (IEP).

BD Pharmingen: The monoclonal antibodies are purified from tissue culture supernatant or ascites by affinity chromatography. The antibodies were conjugated under optimum conditions, and unconjugated antibodies and free dye are removed by gel filtration chromatography. Antibodies are routinely tested by flow cytometric analysis. Other applications were tested at BD Biosciences Pharmingen during antibody development only or reported in the literature.

Biolegend: As knocking out the target protein is one of the most trusted antibody validation processes, we are starting to validate our Cell Biology portfolio antibodies by KO (knockout) and KD (knockdown) systems. To confirm antibody specificity, Western blot data using BioLegend's in-house generated CRISPR/Cas9 and siRNA, as well as CRISPR/Cas9 KO cell lysates from a collaboration with EdiGene (a genome editing company) will be made readily available to researchers
Each lot of antibodies is quality control tested by immunofluorescent staining with flow cytometric analysis. For flow cytometric staining, use the isotype control at the same concentration as your primary antibody.

Absolute Antibody: The 4G2 antibody binds to flavivirus group antigen, protein E. It can be used as an anti- Dengue virus antibody, anti-West Nile virus antibody, anti-Japanese Encephalitis, anti-Yellow Fever Virus or anti-Zika Virus antibody (Aubry et al. 2016) to identify cells infected with these flaviviridae. It binds to the fusion loop at the extremity of domain II of E protein from all four serotypes and prevents syncytia formation (Summers, 1989). The epitope is highly conserved amongst flaviviridae and has been functionally analyzed in detail by Crill and Chang 2004 (PMID: 15564505). Antibody first published in: Nawa et al. Development of dengue IgM-capture enzyme-linked immunosorbent assay with higher sensitivity using monoclonal detection antibody. J Virol Methods PMID:11164919

Eurogentec: Antibodies are validated by purification over protein A or protein G columns depending on the affinity of the immunisation host's IgG. For the purification of culture supernatants, we use generally a protein G column because the protein A has only low affinity for mouse IgG1 subclass which is produced by quite a lot of hybridoma cell lines.

Eukaryotic cell lines

Policy information about cell lines and Sex and Gender in Research

Cell line source(s)	Human HEK293T cells ATCC Cat# CRL-3216<br>TZM-bl NIH Cat#ARP-8129<br>CEM-M7 NIH Cat#ARP-3655<br>U373-MAGI cells- NIH Cat#ARP-3595<br>HMC-3 cells ATCC Cat#CRL-3304<br>ELVIS (BHKICP6LACZ-5) ATCC<br>H4 cells ATCC Cat#HTB-148<br>Vero E6 cells ATCC Cat#CRL-1586
Authentication	ATCC: Short tandem repeat (STR) profiling establishes a DNA fingerprint for every human cell line and may be used as a record of the line. STR profiling, as performed by ATCC, uses multiplex PCR to simultaneously amplify the amelogenin gene and seventeen polymorphic markers, including the most informative polymorphic markers in the human genome. The pattern of repeats results in a unique STR identity profile for each cell line analyzed. The profile can be used as a baseline for comparison with future tests. ATCC uses the Promega PowerPlex 18D system and the ThermoFisher Scientific GeneMapper ID-X v1.2 software for analysis of the amplicons.<br><br>NIH: Tests for bacteria, fungi and mycoplasma were negative.<br><br>Cells were not validated further in our laboratory.
Mycoplasma contamination	Cells were tested routinely to be free of mycoplasma using a PCR based test.
Commonly misidentified lines<br>(See ICLAC register)	No commonly misidentified cell lines were used.

Plants

Seed stocks	N.A
Novel plant genotypes	N.A
Authentication	N.A

ChIP-seq

Data deposition

- [ ] Confirm that both raw and final processed data have been deposited in a public database such as GEO.
- [ ] Confirm that you have deposited or provided access to graph files (e.g. BED files) for the called peaks.

Data access links<br>*May remain private before publication.*	N.A
Files in database submission	N.A
Genome browser session<br>(e.g. UCSC)	N.A

Methodology

Replicates	N.A
Sequencing depth	N.A
Antibodies	N.A
Peak calling parameters	N.A
Data quality	N.A

Software | N.A

Flow Cytometry

Plots

Confirm that:

☐ The axis labels state the marker and fluorochrome used (e.g. CD4-FITC).

☐ The axis scales are clearly visible. Include numbers along axes only for bottom left plot of group (a 'group' is an analysis of identical markers).

☐ All plots are contour plots with outliers or pseudocolor plots.

☒ A numerical value for number of cells or percentage (with statistics) is provided.

Methodology

Sample preparation

To analyze the infection in CEM-M7 cells, three days post-infection, cells were washed with PBS and fixed in 4% PFA. In all the experiments, GFP+ gates were set based on the uninfected cells treated in parallel. Samples were acquired on a CytoFLEX flow cytometer equipped with CytExpert software.

To analyze the expression of markers on macrophage and microglia cells by flow cytometry, cells were detached using Versene (Gibco) and washed using FACS buffer (PBS with 1% (v/v) FBS). For membrane markers, cells were stained with FITC-anti-human P2RY12 Ab (BioLegend, Cat#392107 and isotype Cat#400209), FITC-anti-human CD45 Ab (BioLegend Cat#304005 and isotype Cat#400107), Alexa Fluor® 647 anti-human IBA1 Ab (Abcam, #Ab225261 and isotype #Ab199093), Brilliant Violet 605™ anti-human CD4 Ab (BioLegend Cat#317438 and isotype Cat#400350), APC anti-human CCR5 Ab (BD Pharmingen Cat#550856, isotype Cat#555576), or PE anti-human CXCR4 Ab (BD Pharmingen Cat#551510 and isotype Cat#554689), for 1 h in a wet chamber at RT. Then, cells were washed three times in FACS buffer and fixed in 2% (v/v) PFA. For intracellular markers, cells were permeabilized in 0.2% Triton-X (Sigma-Aldrich) in PBS for 10 min at RT before the addition of antibodies. Samples were acquired on a CytoFLEX flow cytometer equipped with CytExpert 2.3 software. Raw fluorescence-activated cell sorting (FACS) data were analyzed using FlowJo 10.9.0.

Instrument | CytoFLEX flow cytometer (Beckman Coulter)

Software | CytExpert 2.3

Cell population abundance | N/A

Gating strategy | The gating strategy was always the following: all cells SSC-A / FSC-A, single cells FSC-A / FSC-H. For CEM-M7 cells, infected cells (GFP+) positive cells were gates based on uninfected (GFP-) negative cells. Cell marker expression for macrophages and microglia was determined based on antibody vs. isotype staining.

☐ Tick this box to confirm that a figure exemplifying the gating strategy is provided in the Supplementary Information.

Magnetic resonance imaging

Experimental design

Design type | N/A

Design specifications | N/A

Behavioral performance measures | N/A

Acquisition

Imaging type(s) | N/A

Field strength | N/A

Sequence & imaging parameters | N/A

Area of acquisition | N/A

Diffusion MRI ☐ Used ☒ Not used

Preprocessing

Preprocessing software | N/A

Normalization | N/A

Normalization template | N/A

Noise and artifact removal | N/A

Volume censoring | N/A

Statistical modeling & inference

Model type and settings | N/A

Effect(s) tested | N/A

Specify type of analysis: ☐ Whole brain ☐ ROI-based ☐ Both

Statistic type for inference (See Eklund et al. 2016) | N/A

Correction | N/A

Models & analysis

n/a	Involved in the study
☒	☐ Functional and/or effective connectivity
☒	☐ Graph analysis
☒	☐ Multivariate modeling or predictive analysis
